# Combination of Experimental and Bioinformatic Approaches for Identification of Immunologically Relevant Protein–Peptide Interactions

**DOI:** 10.3390/biom13020310

**Published:** 2023-02-07

**Authors:** Jerneja Debeljak, Peter Korošec, Julij Šelb, Matija Rijavec, Mitja Košnik, Mojca Lunder

**Affiliations:** 1 Laboratory for Clinical Immunology and Molecular Genetics, University Clinic of Respiratory and Allergic Diseases Golnik, 4204 Golnik, Slovenia; 2 Faculty of Medicine, University of Ljubljana, 1000 Ljubljana, Slovenia; 3 Faculty of Pharmacy, University of Ljubljana, 1000 Ljubljana, Slovenia; 4 Biotechnical Faculty, University of Ljubljana, 1000 Ljubljana, Slovenia; 5 Allergy Department, University Clinic of Respiratory and Allergic Diseases Golnik, 4204 Golnik, Slovenia

**Keywords:** phage panning, next-generation sequencing, bioinformatic analysis, allergen Ves v 5, epitopes

## Abstract

Protein–peptide interactions are an essential player in cellular processes and, thus, of great interest as potential therapeutic agents. However, identifying the protein’s interacting surface has been shown to be a challenging task. Here, we present a methodology for protein–peptide interaction identification, implementing phage panning, next-generation sequencing and bioinformatic analysis. One of the uses of this methodology is identification of allergen epitopes, especially suitable for globular inhaled and venom allergens, where their binding capability is determined by the allergen’s conformation, meaning their interaction cannot be properly studied when denatured. A Ph.D. commercial system based on the M13 phage vector was used for the panning process. Utilization of various bioinformatic tools, such as PuLSE, SAROTUP, MEME, Hammock and Pepitope, allowed us to evaluate a large amount of obtained data. Using the described methodology, we identified three peptide clusters representing potential epitopes on the major wasp venom allergen Ves v 5.

## 1. Introduction

In recent years, peptides have gained great potential as therapeutic agents due to the advances of new technologies (synthetic, analytic, recombinant) [1] and peptides’ ability to be biophysically and biochemically modified, improving pharmacological properties, such as metabolic stability, cell permeability [2] and solubility [3]. New therapeutic peptides, designed to treat various conditions, and used in oncology and urology to metabolic, cardiovascular and respiratory diseases, can thus be developed [1].

Increased focus is being put onto interfering peptides that inhibit protein–protein interactions (block the protein–protein interaction surface) and thus prevent the downstream signaling events [3]. Management of immunological hypersensitivities such as allergies and autoimmune diseases could thus benefit greatly from the identification of a three-dimensional structure of epitopes, not only due to a better understanding of the immune response to an allergen but also the design of new immunotherapeutic possibilities, where peptides mimicking the epitopes of allergens could occupy the receptors of immune cells and consequently constrain the onset of allergic reaction.

Epitopes are molecular structures recognized by receptors of immune cells (T cells and B cells) and soluble antibodies in the process of immune responses [4]. They can be categorized as either continuous or discontinuous based on whether the residues constituting an epitope are contiguous or not. Continuous epitopes are usually short linear peptide stretches, whereas discontinuous epitopes correspond to several peptide stretches of contiguous residues brought into close proximity by protein folding in 3D space [5]. While all T cell epitopes are linear, B cell epitopes can be either linear or conformational and exposed on the surface of the allergen [6]. Mapping the B cell epitopes on the allergen is especially important for globular inhaled [7] and venom [8] allergens as it has been shown that when such allergens are denatured, their binding capability is significantly reduced [7,8]. In recent decades, various display technologies have been used for the identification of proteins and peptides as well as for in vitro evolution. Thanks to the robustness of filamentous bacteriophage M13, phage display remains the predominant display method compared to others, such as bacterial, yeast or ribosome display [9]. Due to the drop in sequencing costs, phage display can now be combined with next-generation sequencing (NGS), resulting in the capacity for presenting up to 10^6^–10^8^ random peptides that can be evaluated for interaction [10].

Since its development in 1985 [11], phage display technology has been widely exploited for various applications from material sciences [12] to the production of pharmaceuticals [13] and identification of allergen epitopes [14,15,16].

**Figure 1:**
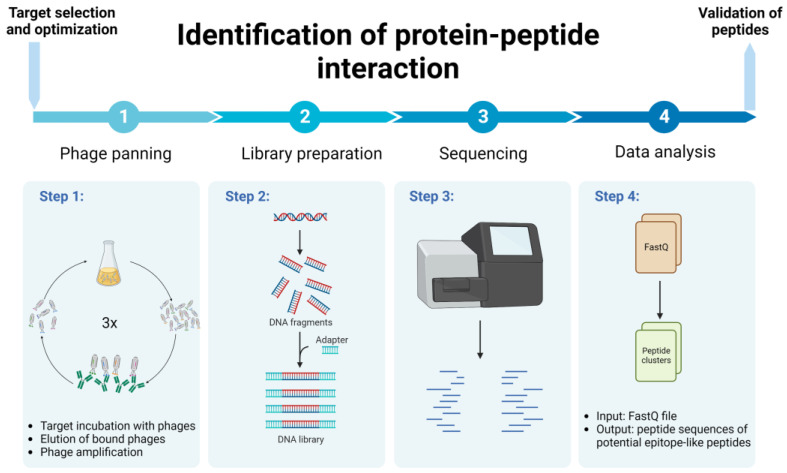
Schematic representation of protein–peptide interaction identification protocol. Created with BioRender.com.

**Figure 2:**
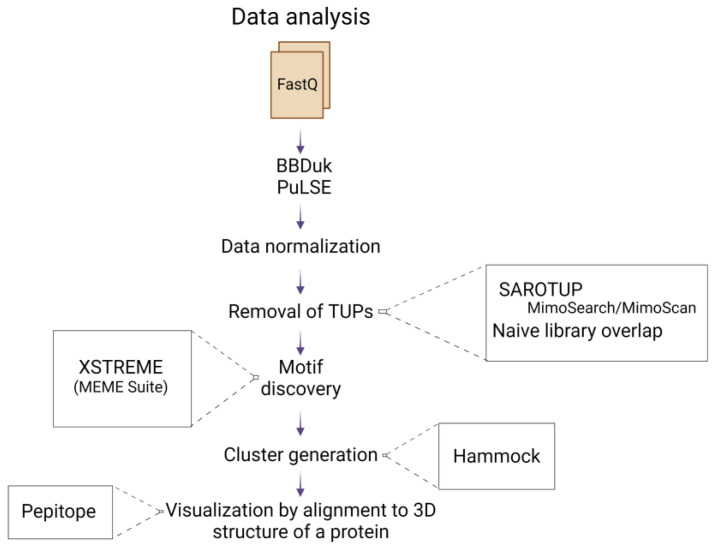
Schematic representation of the data analysis pipeline, starting from the FastQ file input, to the alignment of selected peptides to the 3D structure of the allergen. Created with BioRender.com.

This study presents the methodology for identifying protein–peptide interaction, using a relatively fast, cost-efficient and less labor-intensive protocol, implementing phage biopanning and NGS followed by bioinformatic analysis (Figure 1 and Figure 2). We used the Ph.D. commercial system, which is based on a filamentous M13 phage vector that works as a physical linkage between each variant of a protein sequence and the DNA encoding it [17]. The pipeline for efficient identification of peptide binding partners using various computational tools for detailed data analysis is presented. As an example of the application of this protocol, we present the identification of immunologically relevant allergen Ves v 5 epitopes.

## 2. Materials and Methods

### 2.1. Phage Panning

Three commercial libraries, Ph.D.-12 TM, Ph.D.-7 TM and Ph.D.-C7C TM (New England BioLabs (NEB), Ipswich, MA, USA), were used, consisting of linear 12 or 7 amino acid long peptides or cyclized 7 amino acid long peptides, respectively. The diversity of peptide libraries is 10^9^ peptide variants [17].

Antisera from Ves v 5-immunized rabbits (Thermo Fisher Scientific, Waltham, MA, USA) were affinity purified on protein Ves v 5 (Indoor Biotechnologies, Charlottesville, Virginia, USA), coupled to Dynabeads M-280 Tosylactivated (Thermo Fisher Scientific). Purified polyclonal IgG antibodies were bound to protein G- or protein A-coupled magnetic beads (Dynabeads, Thermo Fisher Scientific). The bead-coupled antibodies were incubated with a phage library for 40 min on a rotating wheel at room temperature. Phage–antibody complex was washed ten times with PBS-T (0.1% in the first panning round and 0.5% in the second and third panning round). The bound phages were eluted with 0.1 M glycine–HCl (pH 2.2), followed by neutralization with 1 M Tris–HCl (pH 9.1) or competitively with 12 µg/mL Ves v 5. Phage amplification occurred in *Escherichia coli* (*E. coli*) host strain K12 ER2738 (NEB) for 4.5 h. After amplification, bacterial cells were centrifuged for 10 min at 12,000× *g*, 4 °C. Phages in the supernatant were further purified according to the manufacturer’s instructions (Ph.D. Phage Display Libraries, Instruction Manual, NEB). Three such biopanning rounds were performed, and after each round, phage DNA from the bacterial pellet after amplification was isolated (GenElute Plasmid MiniPrep Kit, Sigma-Aldrich). DNA concentration and purity were determined using a Thermo Scientific NanoDrop 2000c spectrophotometer.

A naïve library from each of the Ph.D. commercial libraries was also amplified in E. coli followed by DNA isolation.

Sample preparation and next-generation sequencing (NGS).

Fragmentation, end-repair and A-addition were performed with a QIAseq Targeted DNA Custom Panel (QIAGEN, Hilden, Germany), according to the manufacturer’s instructions. For fragmentation, 10 µL 2.5 ng/µL of phage DNA was incubated with fragmentation buffer, 10×, FERA solution and fragmentation enzyme (QIAGEN). The following steps were performed with a QIAseq FX DNA Library Kit. Briefly, adapters and indexes were ligated, followed by library DNA amplification (13 cycles) and purification on magnetic beads. Quantification of final libraries was performed by an Agilent 2100 Bioanalyzer using the High Sensitivity DNA Kit (Agilent Technologies, Santa Clara, CA, USA).

Libraries were normalized to the final concentration of 10 nM and then pooled in equimolar concentrations. The pooled library was loaded into a MiSeq reagent kit v2, run for 300 cycles (Illumina, San Diego, CA, USA) and sequenced by paired-end sequencing on a MiSeq System (Illumina).

### 2.2. NGS Data Analysis

FastQ files were trimmed (BBDuk) in order to obtain high-quality sequences (Phred score > 30). High-quality sequences were subjected to PuLSE software [18] to obtain only the fragments containing the insert of interest (for a detailed description, see Supplementary Materials). For sequences sequenced with reverse primer, reverse complement and translation to amino acid sequence were performed using R (R version 3.6.2). All sequenced reads were normalized prior to further analysis, and the average values of R1 forward primer, R1 reverse primer, R2 forward primer, R2 reverse primer were used. Boman indices were calculated using the Peptides package (R, version 3.6.2). The Boman index gives an overall estimate of a peptide’s binding potential to other proteins, based on the amino acid sequence. A high binding potential of a peptide is indicated when the index value is higher than 2.48 [19]. For the detection of target-unrelated peptides (TUPs), SAROTUP (MimoSearch and MimoScan) [20] was used. Additionally, libraries after selection were compared with an amplified naïve library in order to detect any peptide sequences present in both libraries. To find repetitive motifs, sequences were imported into MEME Suite-XSTREME [21], where sequences from the naïve library were used as a background. Default parameters, with the exception of minimum width, which was 3, were used. Heatmaps of amino acid profiles were made by the heatmaply package (R, version 3.6.2). Target binding peptides from three libraries were combined and subjected to the Hammock tool [22] for cluster generation. Default parameters in “full” mode were used, with the exceptions of “--min_conserved_positions”, which was set to 3, “--max_shift”, which was set to 1, and “--assign_thresholds”, which was set to 10.0, 8.0, 6.75. These values were set empirically. Logos were created by WebLogo. Sequences containing a motif were subjected to Pepitope [23] to obtain predicted regions of peptide alignment on the allergen Ves v 5 (PDB: 1QNX). Alignment of best clusters was visualized in PyMol.

### 2.3. Microarray Analysis

A total of 36 sera from individuals with wasp venom- and Ves v 5-specific IgE antibody (sIgE) levels higher than 0.35 kU/L (quantified using Immulite 2000 XPi, Siemens Healthcare GmbH, Germany) were subjected to microarray assay (JPT Peptide Technologies, Berlin, Germany). As there is a possibility of high intervariability of IgG antibodies among individuals, six patient sera were combined into one sample, resulting in total of six samples. Briefly, 83 peptides identified in this study (linear and their respective cyclic form) were immobilized on a glass slide (JPT Peptide Technologies) with a hydrophilic linker moiety trioxatridecan-succinamic acid (Ttds) and incubated with patient sera, and diluted 1:200 in blocking buffer (Pierce International, Superblock TBS T20) for two hours at 30 °C. Bound antibodies from the sera were detected with secondary, fluorescently labeled AlexaFluor647-anti-human-IgG antibodies at 0.1 µg/mL, which were left to interact for 1 h. False positive binding to peptides was performed by applying the secondary antibodies directly to the immobilized peptides, without samples. Fluorescence intensity profiles were obtained by scanning the slides with a high-resolution laser scanner at 635 nm. Finally, by quantification of slide images, a mean pixel value for each peptide was yielded. Before data interpretation, the signal intensities of false positive binding for respective peptides were subtracted from the signal intensities of individual samples. For the interpretation of the results, the median of all intensity signals was calculated. The difference in signal intensities between linear and cyclic peptides was calculated using the Wilcoxon rank sum test, as the same peptides in two different forms were compared (on the same groups of individuals) and the data were non-normally distributed.

## 3. Results

### 3.1. Elimination of Non-Target Binders

Two independent selection pressures define which peptides will be abundant in the final output. Sequential rounds of biopanning result not only in the amplification of target binders but also in the amplification of non-specific, so-called target-unrelated peptides (TUPs). TUPs bind to other components used during the panning procedure [24]. The other selection pressure appears during the amplification step, since peptides display different amplification rates, and the ones with advantageous amplification rates in E. coli will become more abundant [25]. Features of such peptides are positive net charges, low hydrophobicity or high Boman indices [26]. This means that non-target binders will always be present in the sample obtained after the experimental procedure and need to be identified during the bioinformatic analysis.

In this study, we used three commercial libraries; Ph.D.-12, Ph.D.-7 and cyclic Ph.D.-C7C (New England BioLabs) [17]. After three panning rounds, we subjected peptide-presenting bacteriophage DNA sequenced by NGS to PuLSE software [18], which produced 100 of the most abundant unique sequences of each panning round.

**Figure 3:**
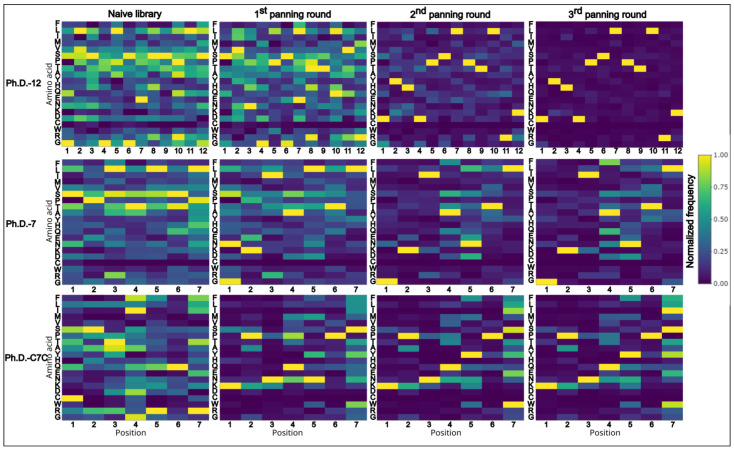
Heatmaps of normalized data from amino acid frequencies of all sequences. Amino acid distribution of normalized data shows positional enrichment of amino acids. Clear shift towards reduced diversity can be seen with successive rounds of panning. Ph.D.-12 is highly affected by the dominant sequence that appears in two-thirds of all sequenced peptides, thus, the overall diversity drops significantly. On the x-axis positions of amino acids are plotted and on the y-axis the respective amino acids are listed in the following order (top to bottom): F, L, T, M, V, S, P, T, A, Y, H, Q, E, N, K, D, C, W, R, G.

When examining the amino acid composition of the libraries, and how it changes through panning rounds, an apparent reduction of amino acid diversity with each consecutive panning round can be observed (Figure 3). Certain amino acids become underrepresented during the selection process, while others appear overrepresented (Figure 3). Strong positional overabundance of specific amino acids can be seen in panning rounds of Ph.D.-12. Moreover, even in amplified naïve libraries, without selection pressure, the amino acid composition is affected by the amplification bias.

To eliminate TUPs, two approaches were taken. First, unique peptide sequences from libraries of each panning round were subjected to MimoScan and MimoSearch (SAROTUP) in order to obtain sequences that have previously been reported by other biopanning studies (Supplementary Table S1). All tools within the SAROTUP web server are based on the BDB database and are freely available [20].

Additionally, we also compared the peptides from libraries after selection with an amplified naïve library. Sequences present in both libraries were regarded as TUPs. All sequences identified as TUPs with either of the two approaches were eliminated from further analysis.

After each panning round, a rather significant drop in TUPs is evident in Ph.D.-12, while in Ph.D-7, the drop is more gradual (Table 1), resulting in 8.7% and 13.4% of TUPs present in the final output, respectively. Cyclic library Ph.D.-C7C does not appear prone to target-unrelated binding since we only obtained 0.5% of TUPs present in the first panning round and 2.7% after the third panning round (Table 1).

Amplification is considerably prioritized for DYHDPSLPTLRK (63.3% of the whole library after the third panning round). This has also been noticed by Juds et al., where they report DYHDPSLPTLRK and GNNPLHVHHDKR as the most abundant sequences in NGS as well as Sanger pools while screening for poly(propylene) binders [26]. This coincides with our data, where we detected the two peptides in high copy numbers in NGS pools (Table 2). Among the 20 most abundant peptides, other TUPs appear; however, this happens only in linear libraries and none in the cyclic library. On average, peptides from the cyclic library also exhibit lower Boman indices, which is likely the consequence of two cysteines in the sequence (Table 2).

### 3.2. Amino Acid Motifs of Potential Conformational Epitopes

**Figure 4:**
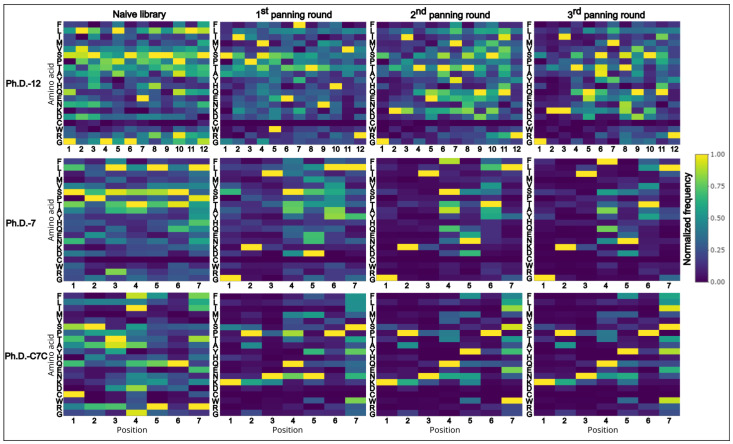
Heatmaps of normalized data from amino acid frequencies from the cleaned data (unique sequences without TUPs). On the x-axis positions of amino acids are plotted and on the y-axis the respective amino acids are listed in the following order (top to bottom): F, L, T, M, V, S, P, T, A, Y, H, Q, E, N, K, D, C, W, R, G.

From the collection of peptide sequences acquired from the cleaned data (unique sequences without TUPs) (Figure 4), we determined the common motifs which appear repetitively among the sequences. This approach allows us to identify the consensus sequence that comprises a protein–peptide interaction site (epitope-like peptide in antigen–antibody binding). All the sequences from each panning round were subjected to XTREME software [21], a part of MEME Suite [27].

**Figure 5:**
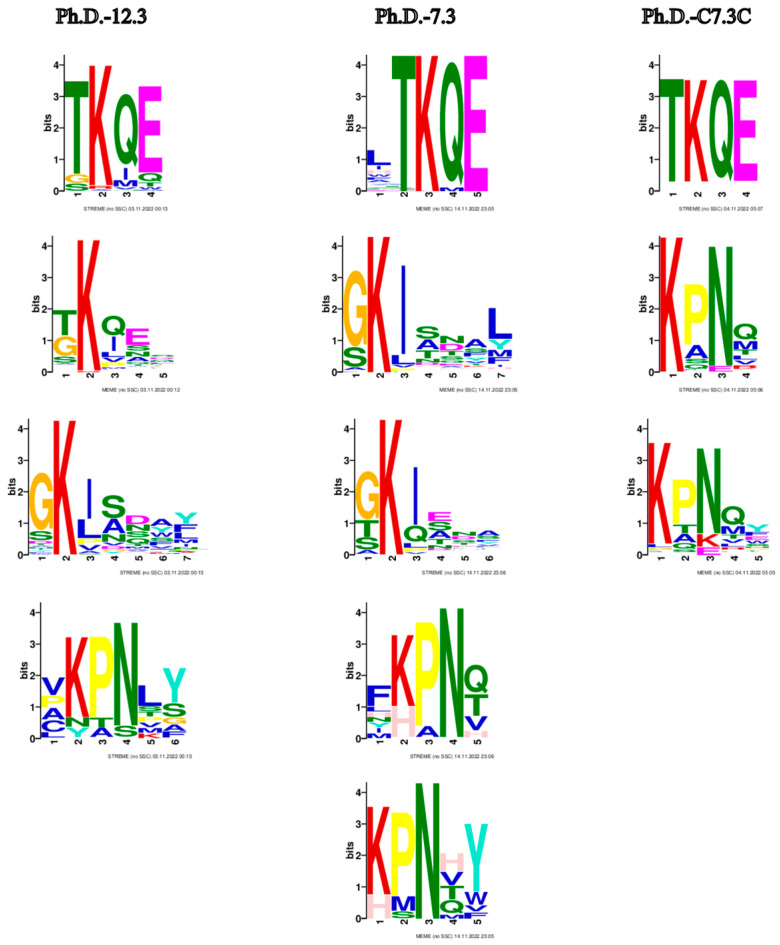
Motifs from biopanning rounds of three phage display libraries. Common motifs shared among the libraries appear to be TKQE, GKI and KPN. Only motifs with *p* < 0.05 are represented. Default parameters with the exception of minimum width: 3 were used. The enriched motifs are the potential constituents of epitope-like peptides.

The most prominent consensus sequences based on all three libraries are TKQE, GKI and KPN (Figure 5). These motifs already appear after the first panning round (Supplementary Figure S1).

We have also searched the IEDB website for any previously identified peptides containing a motif that we identified in this study. Currently, 42 records for Ves v 5 epitopes are listed in the IEDB database (https://www.iedb.org/, accessed on 10 November 2022). One epitope harbors the TKQE motif: VVSYGLTKQEKQ, and one only partially: NKVVVSYGLTKQ. There are also three epitopes with a KPN motif: ACKYGSLKPNCG, LKPNCGNKVVVS, YGSLKPNCGNKV, none of which would contain a GKI motif. However, all currently identified epitopes in the IEDB database are obtained through T cell assays.

### 3.3. Identification of Three Peptide Clusters Indicates Three Potential Epitope Regions on the Allergen

**Figure 6:**
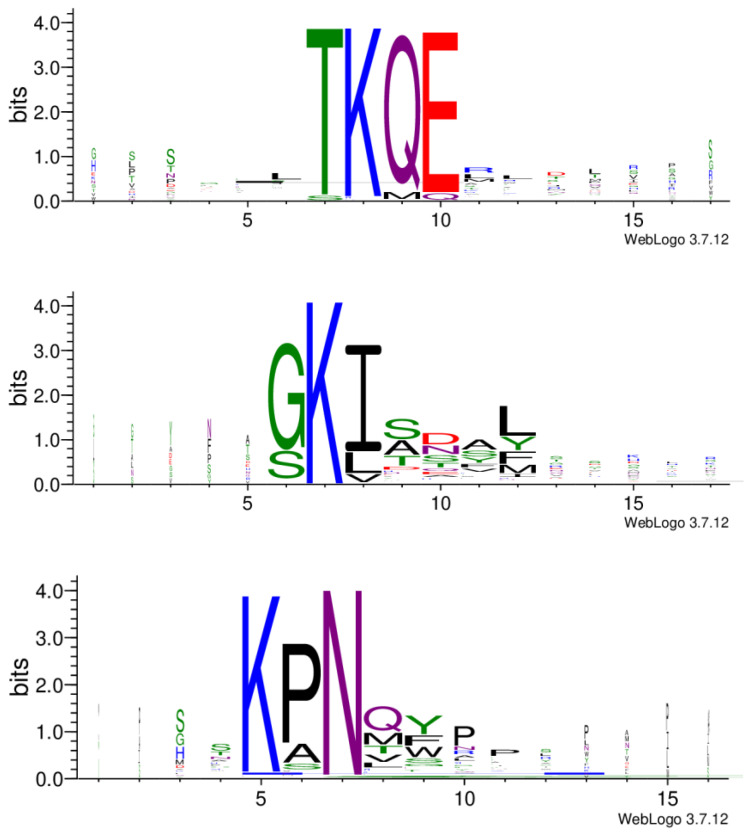
Three peptide clusters generated by Hammock. Five hundred and twenty-five peptides were subjected to the Hammock tool. Sequence logos, representing the significance of the individual amino acid residues were created with WebLogo.

In further analysis, all the cleaned data from the three libraries were combined, and the resulting 525 peptides were subjected to the Hammock tool [22], which produced three clusters of the most similar peptides (Supplementary Table S2). WebLogo [28] created the logos of sequences in each cluster (Figure 6). The generated clusters were based on the same motifs we observed before with the MEME Suite. In the KPN motif, proline can be substituted with alanine or, less frequently, with serine. In position 8 (after asparagine), an amino acid with a polar side chain (glutamine or threonine) or non-polar side chain (methionine, valine or leucine) appears. This is then followed by an aromatic amino acid (tyrosine, phenylalanine or tryptophan). Motif GKI almost exclusively appears at the beginning of the sequence. Glycine may be substituted with serine, and isoleucine with leucine or valine. Serine is also often present in positions 9 to 11. Position 12 may be occupied with non-polar leucine or methionine, or aromatic tyrosine or phenylalanine. Amino acids in motif TKQE appear rather dominant without other possible sequence variations (Figure 6).

**Figure 7:**
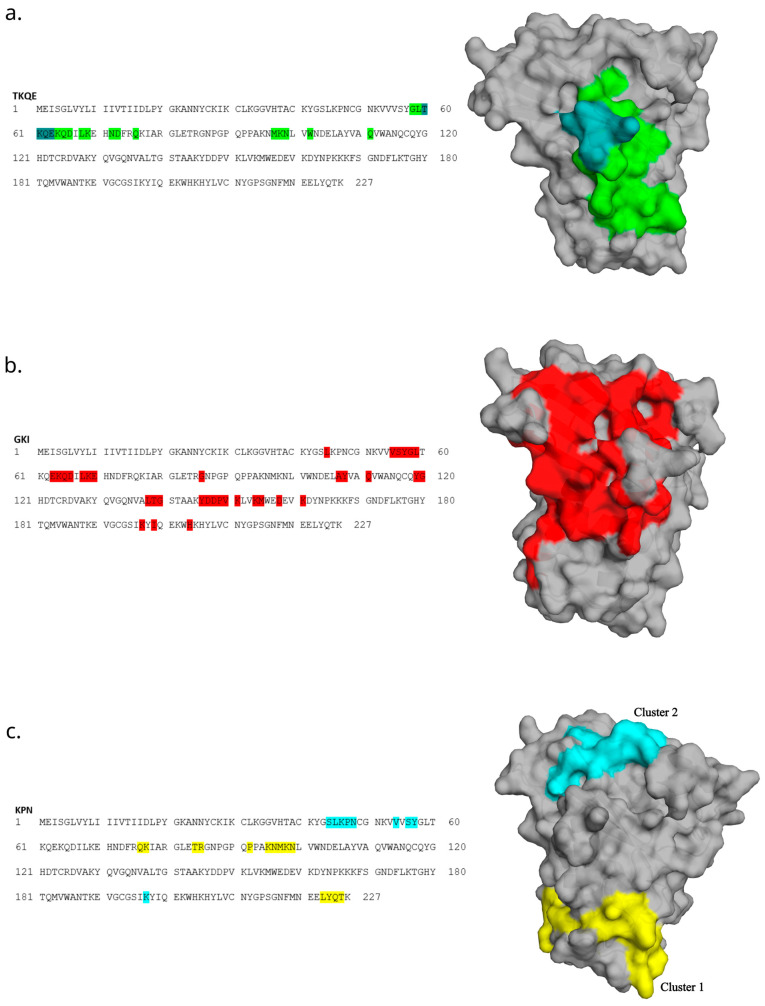
Mapping the top 20 most abundant epitope-like peptides to the primary and 3D structure of allergen Ves v 5. Mapping of TKQE- (**a**), GKI- (**b**) and KPN-containing (**c**) peptides was performed in the Pepitope tool and is based on the corresponding motifs. The strongest cluster representing the predicted epitope region was selected and is presented as the colored area on the allergen structure. For the KPN motif, the two strongest clusters are presented. Three-dimensional models of allergens were created with the PyMOL Molecular Graphics System, Version 2.5.2, Schrödinger, LLC.

Motif-containing peptides were then mapped onto a 3D model of allergen Ves v 5 using the Pepitope tool [23]. With this approach, we could obtain the predicted areas of the allergen that bind to an antibody. (Figure 7). The best clusters are presented graphically.

The predicted cluster of motif TKQE involves the residues of linear sequence TKQE, which are located on a very exposed stretch of the structure (Figure 7a). The predicted cluster of the motif GKI appears to cover a relatively large stretch of the structure and should thus be interpreted with consideration (Figure 7b). The discontinued cluster of the KPN motif does not include the linear sequence KPN that is present in the primary sequence (Figure 7c, yellow). Thus, we also graphically present the second-best cluster, which involved the KPN residues (Figure 7c, turquoise).

### 3.4. Binding of IgG Antibodies to Epitope-like Peptides

Following in silico prediction, we continued to see how well the selected peptides bind to IgG antibodies from sera of Ves v 5-sensitized individuals.

We analyzed the sera of 36 subjects with high sIgE for Ves v 5 as we presume they also have high IgG antibodies. Six patient sera were pooled into one sample, resulting in a total of six pooled samples (Supplementary Table S3).

We have chosen 83 peptides for microarray analysis (Supplementary Tables S4 and S5). Of those, 80 peptides were present in the final output after three panning rounds, and three were only present in the naïve library and were used as a negative control.

A threshold for selection of peptides was set to the peptide’s fluorescence intensity signal that was at least twice as high as the strongest negative control, as it results in a reasonable number of peptides for further chemical synthesis and in vitro studies, as the potential epitope-like peptides (Table 3). These peptides originate from all three Ph.D. libraries and represent all three identified motifs.

IgG antibodies in all six sera-containing samples bind significantly better to cyclic peptides than to the relevant linear peptides (*p* < 0.001) (Table 4).

## 4. Discussion

Here, we present a protocol for protein–peptide interaction identification, where single or multiple interaction sites are predicted. The methodology can be utilized for various applications where the binding of a target protein to short peptides is of interest. As an example of the application of this protocol, we present the identification of allergen Ves v 5 epitopes.

The phage display technology, in combination with NGS, became a powerful tool available to laboratories and research institutions worldwide. Newly available bioinformatic tools for phage data analysis make the entire process of binding interaction identification even more accessible. As different tools, specifically designed to process large amounts of data produced by phage panning and NGS, are used, no extensive computational skills are required to perform analyses. Hence, the pipeline can be employed by various laboratories, even if they are predominantly wet lab oriented. Additionally, these bioinformatic tools allow us to analyze data in a way that can be tailored to our specific needs. The presented tools are freely available and can be considered a valuable resource for future experiments.

The recently designed open-source tool PuLSE proved very practical for extracting the peptide sequences from the entire data of sequenced reads in a FastQ file.

Iterative rounds of selection by washing off TUPs followed by enrichment of target binders and iterative rounds of performing incubations on protein A- and protein G-coupled beads succeeded in decreasing the amount of TUPs (Table 1) but did not completely eliminate them.

We show that by combining SAROTUP tools (removing material binders) and comparing sequences after selection with sequences from an amplified naïve library (removing peptides with propagation advantages), a significant amount of noise can be removed. In addition, SAROTUP is based on BDB, and thus with new experiments, more sequences will be uploaded, therefore, more of the TUPs will likely be detected. A combination of both approaches is recommended, as identified peptides by the respective approaches do not entirely overlap. Comparison of peptides after selection with the naïve library finds more peptides; however, it should not be disregarded that some of these peptides may display binding to the target while at the same time show strong propagation preferences. Due to a large amount of data, eliminating such peptides should not affect the final output.

In none of the three libraries (during either of the selection steps) did we detect more than 30% of parasitic sequences. Interestingly, in the cyclic library, we detected less than 3% of TUPs, indicating that cyclized peptides may have higher specificity for target binding. As such, it is a valuable addition to linear libraries.

Based on the motifs from XSTREME software, even one panning round successfully selects peptides with motifs that continue to appear throughout the panning process.

Here, we present three key motifs, obtained from the first selection round to the third, for all three libraries. We show that KPN and TKQE are motifs contained not only within T cell but also within B cell epitopes and are thus exposed on the allergen surface. The IEDB database shows no identified Ves v 5 epitopes containing the GKI motif, which could indicate that this motif appears only as a part of a conformational epitope and not as linear (as only linear epitopes are currently listed in IEDB).

When aligning the peptides onto the 3D structure of the protein, each result should be evaluated separately and interpreted in relation to the primary structure.

The current field of research is in high demand for faster ways of mapping the exact location of a molecule’s interaction with its interacting partner. Reliable tools producing accurate results for localizing interaction sites will be a valuable resource in the future.

From 15 amino acids constituting an epitope, about five significantly influence binding, while the rest only impact the binding to a certain extent (max. up to one order of magnitude) [29]. This could explain why we obtain motifs with three to four very strong amino acids while the rest appear weaker. Furthermore, antibodies raised against a particular epitope may display stronger binding to a mutated epitope [29], indicating that with the presented pipeline, we could obtain peptides with even stronger affinity compared to the natural epitopes of the antibodies, which is a promising starting point in the design of therapeutic peptides.

We performed a peptide binding assay to test for the binding of selected peptides to the IgG antibodies in the sera of Ves v 5-sensitized individuals. Due to the possible intervariability of IgG antibodies among individuals, we suggest the pooling of the samples in order to detect the potential immunodominant peptides. Based on the intensity of the fluorescent signal, we prioritized a set of 18 linear and 7 cyclic epitope-like peptides, where 6 of them appear in both peptide sets (Table 3). These peptides can then be used in the following in vitro experiments using the sera of each individual separately.

Strictly based on in silico data, the TKQE motif would appear as the most prominent potential epitope; however, IgG binding shows strong interaction with TKQE motif-containing peptides as well as with GKI and KPN motif-containing peptides, which implies further testing of peptides with either of the three motifs.

In addition to the previously observed unique behavior of the cyclic library, and also comparing the binding of cyclic vs. linear peptides on the microarray, a significant increase in binding to cyclic peptides is observed (*p* < 0.001) (Table 4). With this data, we further support the evidence of higher binding specificity of cyclized peptides.

A vast amount of bioinformatic tools designed in recent years, specifically for phage panning data analysis [18,20,22,23,27,30,31], indicate that phage display followed by NGS and data analysis for identification of protein–peptide interactions is a prosperous field continuously developing and improving.

## 5. Conclusions

With this study, we aimed to present an applicable protocol for the identification of various protein–peptide interactions. The above-described tools help us to prioritize the peptides used in further in vitro experiments that should be performed to validate the data obtained in silico.

## Figures and Tables

**Table 1:** Removal of target unrelated peptides (TUPs) through the selection process. With each panning round, the percentage of TUPs decreases in linear libraries, whereas the cyclic library results in the lowest percentage of TUPs after the second panning round where, in fact, no TUPs were detected.

	Nr. of All Unique Sequences	Nr. of Unique TUPs	%
Ph.D.-7			
7.1	207	55	25.6
7.2	195	36	18.5
7.3	202	27	13.4
Ph.D.-12			
12.1	209	62	29.7
12.2	201	34	16.9
12.3	184	16	8.7
Ph.D.-C7C			
C7.1C	207	1	0.5
C7.2C	170	0	0
C7.3C	187	5	2.7

**Table 2:** List of 20 most abundant sequences from each Ph.D. library after the third panning round. Sequences and their abundances were obtained by the PuLSE software. Sequences highlighted in gray also appeared in naïve library or BDB database (detected by SAROTUP tools) and are regarded as TUPs. All data were normalized prior to analysis. Boman indices for peptides from the cyclic library were calculated with the addition of one cysteine on each end of the peptide. A peptide has high binding potential if the Boman index value is higher than 2.48. Occ.—occurrence.

	Ph.D.-12	Occ.	%	Boman Index	Ph.D.-7	Occ.	%	Boman Index	Ph.D.-C7C	Occ.	%	Boman Index
1	DYHDPSLPTLRK	111,085	63.30	3.237	GKIFNTL	18,843	16.05	0.143	KPNQYPI	13,587	10.04	1.154
2	GKITQAMNVSQR	4597	2.62	2.508	GKIADLG	8971	7.64	0.106	KANQFPW	8520	6.30	0.894
3	STKQETYTDKHY	4522	2.58	4.018	NERALTL	8018	6.83	2.756	SGKPNVW	4130	3.05	0.636
4	QVNGLGERSQQM	3429	1.95	2.933	YSLQSVI	6743	5.74	−0.2	LDKPNRY	3895	2.88	3.166
5	RDYHPRDHTATW	2546	1.45	4.812	GYKDFSA	4736	4.03	1.726	KANMWPS	3642	2.69	0.727
6	TAKYLPMRPGPL	2047	1.17	0.687	FGHYHYA	3130	2.67	0.553	NKPNQYF	3431	2.54	2.108
7	GKIVDSLGQSSP	1652	0.94	1.188	TNAWVDG	2006	1.71	1.259	KPNQSNY	3214	2.38	2.817
8	VKPNLYPSNDPI	1410	0.80	1.434	SITPMPA	1985	1.69	−0.444	SHKPNVF	2588	1.91	1.186
9	GKISDRIKFDDG	1314	0.75	3.407	TPARHIY	1831	1.56	2.223	KANKYPS	2152	1.59	1.879
10	MGTKQEHLGPIR	1313	0.75	2.165	YKANQFL	1769	1.51	1.166	KPNMYPL	1997	1.48	0.278
11	HMETKQEKQIIW	1259	0.72	2.376	AHKSNHY	1597	1.36	3.320	KPNVYPL	1722	1.27	0.090
12	AEMTKQESILQR	1213	0.69	3.095	GKIDYFI	1428	1.22	0.093	KANQERT	1699	1.26	4.184
13	NWTKQERWAVSA	1034	0.59	2.759	FHPNTYN	1313	1.12	2.524	TKQEGRT	1697	1.25	3.829
14	GNNPLHVHHDKR	997	0.57	3.879	SPSTHWK	1211	1.03	2.464	KANSFGS	1632	1.21	1.189
15	DPKPNSSDYWYF	995	0.57	2.617	LSNNNLR	1173	1.00	4.057	KPNQFPR	1599	1.18	3.012
16	LPAHTKQEMRYL	921	0.52	2.182	VKPNQYA	1162	0.99	1.717	KPNQYPT	1528	1.13	1.987
17	SKIETSLNSMTN	909	0.52	2.399	GKIDSYF	949	0.81	1.281	SKPNMYS	1521	1.12	1.580
18	ITKQEALDTQIR	905	0.52	2.970	SKPNVYW	833	0.71	1.337	KPNLYPY	1511	1.12	0.554
19	HTKSNQWYPFQM	905	0.52	2.198	GHKMNHY	804	0.69	2.623	KENQWPS	1392	1.03	2.561
20	GKIGQYFSEYAT	799	0.46	1.047	GKITSMY	775	0.66	0.143	KPNVFPS	1378	1.02	0.668

**Table 3:** Peptides studied by microarray with the signal intensity twice as high as the strongest negative control. Left: linear peptides, right: cyclized peptides.

	Sequence	Median Signal Intensity		Sequence	Median Signal Intensity
1	HTKQELL	42,935.17	1	CGFKPNMFYYPELC	39,741.17
2	YSSLKPNKYAVW	32,754.17	2	CYSSLKPNKYAVWC	32,991.50
3	GFKPNMFYYPEL	26,880.67	3	CDPKPNSSDYWYFC	29,583.17
4	KANQFPW	25,764.67	4	CKANQFPWC	27,706.17
5	GKIGSFLGGGHI	23,977.83	5	CGKIDSYFC	26,333.83
6	DPKPNSSDYWYF	23,049.67	6	CVKPNLYPSNDPIC	24,513.67
7	GKIDSYF	21,866.83	7	CGKIDSFIRVEHGC	23,558.17
8	IAHKPNQGWWIH	20,982.33	CTRL	CQQLNIPPC	11,382.17
9	GFAGKIASTFVD	20,143.00	CTRL	CAHRVQTAC	4026.17
10	GKIDYFI	19,626.83	CTRL	CNLLMSHAC	3364.50
11	QGKPNQWANYFL	17,935.50			
12	HMETKQEKQIIW	16,990.83			
13	GKIDSFIRVEHG	16,587.00			
14	GKIGQYFSEYAT	16,452.83			
15	GKISSVMAHGDW	15,244.00			
16	STKQETYTDKHY	15,017.33			
17	LLANTGKIQKYL	14,572.50			
18	TKQELPY	13,254.17			
CTRL	QQLNIPP	6598.50			
CTRL	AHRVQTA	3720.00			
CTRL	NLLMSHA	3311.83			

**Table 4:** IgG antibodies from the sera bind significantly better to the cyclic form of peptides compared to their respective linear form. Signal intensities of serum pools were compared between the two peptide forms. Wilcoxon rank sum test was performed.

	#1	#2	#3	#4	#5	#6
*p* value	<0.0001	0.0003	<0.0001	<0.0001	<0.0001	<0.0001

## Data Availability

The data supporting the findings of this study are available in the Supplementary Materials and from the corresponding authors upon reasonable request.

## Supplementary Materials

The following supporting information can be downloaded at: https://www.mdpi.com/article/10.3390/biom13020310/s1, Table S1: Sequences from the third round of panning appearing in other (previously reported) biopanning experiments, obtained from SAROTUP web server; Table S2: Three peptide clusters generated by Hammock; Table S3: Demographic and serologic features of individuals included in the study; Table S4: Sequences of linear peptides subjected to microarray analysis and the respective immunofluorescence intensities from six groups of individuals; Table S5: Sequences of cyclic peptides subjected to microarray analysis and the respective immunofluorescence intensities from six groups of individuals; Figure S1: Motifs obtained after the first and second panning round, obtained from the XSTREME tool.
