# Supplementary material for: Combination of Experimental and Bioinformatic Approaches for Identification of Immunologically Relevant Protein–Peptide Interactions
Source: Biomolecules. 2023 Feb 7;13(2):310. doi: 10.3390/biom13020310 (PMC9953301; doi:10.3390/biom13020310)
Supplement: Supplementary file 1 [file biomolecules-13-00310-s001.zip › biomolecules-2183779-supplementary.pdf]

ONLINE SUPPLEMENT

COMBINATION OF EXPERIMENTAL AND BIOINFORMATIC APPROACHES FOR IDENTIFICATION OF IMMUNOLOGICALLY RELEVANT PEPTIDE-PROTEIN INTERACTIONS

Input parameters for PuLSE software:

Insert of interest was identified by the software through the determination of flanking sequences.

For linear library (Ph.D.-7 and Ph.D.-12):

Fw: CAC TCT $(XXX)_{7/12}$ GGT GGA

Rv: TCC ACC $(XXX)_{7/12}$ AGA GTG

For cyclic library (Ph.D.-C7C):

Fw: GCT TGT $(XXX)_7$ TGC GGT

Rv: ACC GCA $(XXX)_7$ ACA AGC

At the end of the PuLSE script, for forward direction, an argument "UAG Q" was added to change the coding from a stop codon into glutamine (in phage expression system modification is made, so that UAG instead of coding a stop codon, it expresses a glutamine residue).

Elimination of non-target binders

Suppl. Table 1: Sequences from the third round of panning appearing in other (previously reported) biopanning experiments. Data was analyzed using MimoScan and MimoSearch. We obtained sequences that have already appeared in previous panning experiments (thus, likely not target binders). Accessed on 10.11.2022

Query	Matched Peptide	Biopanning Data Set ID	Target Name
**Ph.D.-12**			
DYHDPSLPTLRK	DYHDPSLPTLRK	3260	Anti-imidaclothiz monoclonal antibody 1E7
	DYHDPSLPTLRK	3517	P. aeruginosa H103 (PAO1 wild-type prototroph)
QVNGLGERSQQM	QVNGLGERSQQM	3376	Complement decay-accelerating factor
	QVNGLGERSQQM	3448	IgE from patients with allergy to Scylla paramamosain AK
	QVNGLGERSQQM	3517	P. aeruginosa H103 (PAO1 wild-type prototroph)
RDYHPRDHTATW	RDYHPRDHTATWGGG	3109	NIST RM8670 (NISTmAb)
SPLRAVAFSGAQ	SPLRAVAFSGAQGGG	3109	NIST RM8670 (NISTmAb)

VCSPCGPVPPAK	VCSPCGPVPPAKGGG	3109	NIST RM8670 (NISTmAb)
GADTSKPPRFVT	GADTSKPPRFVTGGG	3109	NIST RM8670 (NISTmAb)
GNNPLHVHHDKR	GNNPLHVHHDKRGGG	3109	NIST RM8670 (NISTmAb)
	SGNNPLHVHHDKR	3211	T lymphocyte leukemia cell line Jurkat
	GNNPLHVHHDKR	3387	Endoglin
**Ph.D.-7**			
NERALTL	NERALTL	2191	Epoxy
	NERALTL	2978	Fusion glycoprotein F0
	NERALTL	3013	Fusion protein
	NERALTL	3257	Human prostate cancer cell line PC3
	NERALTL	3327	Anti-Haemonchus contortus polyclonal antibody
	NERALTL	3330	Beta-nerve growth factor, Beta-NGF
GYKDFSA	GYKDFSA	2977	Hemagglutinin-esterase (HE)
	GYKDFSA	3012	Hemagglutinin-esterase, HE
TPARHIY	TPARHIY	2978	Fusion glycoprotein F0
	TPARHIY	3013	Fusion protein
	TPARHIY	3257	Human prostate cancer cell line PC3
SPSTHWK	SPSTHWK	2413	Crystalline Ni3B
TFASHDQ	TFASHDQ	3330	Beta-nerve growth factor, Beta-NGF
TNSSFHK	TNSSFHK	2412	Amorphous Ni3B
LGSPMSN	LGSPMSN	3330	Beta-nerve growth factor, Beta-NGF
MTKQEYA	CTGRMTXQXXXA	358	
HWSTTIS	HWSTTIS	3330	Beta-nerve growth factor, Beta-NGF
RILITIP	RILITIP	2977	Hemagglutinin-esterase, HE
	RILITIP	3012	Hemagglutinin-esterase, HE
MTKQEAL	CTGRMTXQXXXA	358	
MTKQETM	CTGRMTXQXXXA	358	
LSNNNLR	LSNNNLR	2349	Silicon dioxide, SiO2
	LSNNNLR	3257	Human prostate cancer cell line PC3

Three sequences containing the TKQE motif recognized by MEME software, were found in the SAROTUP web server tools, since they contained a consensus MTXQX and were thus also removed from further analysis.

The principle of the SAROTUP web server is that a peptide which is not found in the BDB database might be a specific binder to the target of interest. On the other hand, if a peptide is found in BDB (and obtained through a different target), it is more plausible to be noise since there is a small chance of obtaining identical peptides by different targets (http://i.uestc.edu.cn/sarotup3/cgi-bin/MimoSearch.pl access: 10.11.22).

At this point, SAROTUP PhD7Faster 2.0 can also be used to look at the Ph.D.-7 and Ph.D.-C7C sequences that have a growing advantage during replication and are thus less likely to be specific binders.

Amino acid motifs of potential conformational epitopes

Suppl. Figure 1: Motifs obtained after the first and second panning round, obtained from the XSTREME tool. Common motifs TKQE, GKI and KPN, can already be observed after the first panning round.

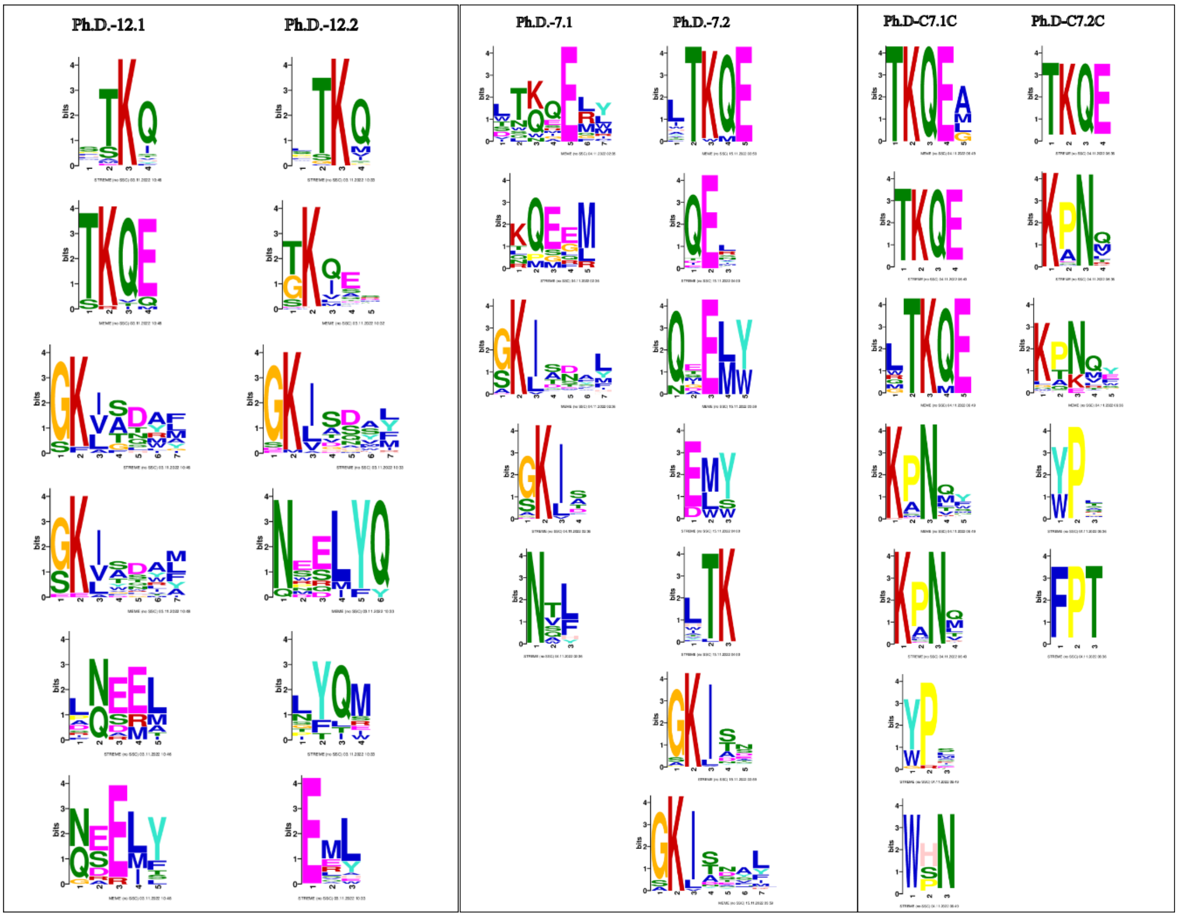

Identification of three peptide clusters indicates three potential epitope regions on the allergen

Suppl. Table 2: Three peptide clusters generated by Hammock. Cleaned data of 525 peptides were subjected to the Hammock tool. 460 peptides were distributed into clusters.

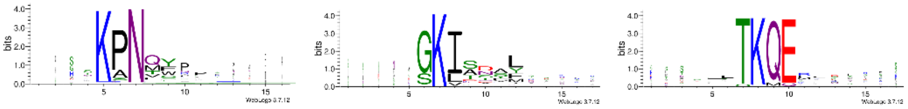

YSSLKPNKY-AVW---	---YAGKISVGMRGH--	-----YTKQEVW-----
--YNKPNHY-------	----TSKIADFYGFKN-	-----WTKQESL-----
---YKPNVY-V-----	-TDGRGKIYSWFS----	---WSKTKQE-------

---YKLNQWPTPLMP-	-SYSQGKIAASFP----	---WPWTKME-------
---YKANQFL------	--SSNGKVSNYFVY---	WNNPQFTKQEMI-----
---WKPNQA-PFPAPM	-----SKVADWF-----	---WGMTKQE-------
VPKPNVGMWGPV----	-----SKLADYL-----	VTSTLGTKQEVV-----
---VKPNQY-M-----	-----SKLADFL-----	-----VTKQEYM-----
---VKPNQY-A-----	-----SKLADAL-----	--VQNGTKQELLMT---
---VKPNLY-PSWNMS	-----SKIYTSL-----	---VPLTKQQALDQG--
---VKPNLY-PSNDPI	-----SKIYDYNTSHGV	-----TTKQERL-----
----TVGSWMDNKVIA	-----SKITTKL-----	-----TTKQEQISMSSS
-----TNAWVDG----	-----SKITSVL-----	TPTGWTTKQERV-----
---TKSNQYQ------	-----SKITQYF-----	---TPPTKQESYLMR--
---TKSNMYN------	-----SKITNFY-----	--TNVYTKQESMIM---
---TKPNQTQ------	-----SKITDYL-----	------TKQERYS----
---TKPNQSS------	-----SKISSWY-----	------TKQERPQ----
---TKPNQSI------	-----SKISNYM-----	------TKQERPL----
---TKPNQSA------	-----SKISDWF-----	------TKQERAV----
---TKPNQGH------	-----SKISDTYGWKVW	------TKQEMRL----
---TKLNMYN------	-----SKISDSF-----	------TKQEMHY----
---TKANQFR------	-----SKIQDVL-----	------TKQELPY----
--STKPNTF-------	-----SKIHDYLGEQLK	------TKQEHST----
--STKPNQS-------	-----SKIGDAL-----	------TKQEGRT----
--STKPNQF-------	-----SKIETSLNSMTN	------TKQEGNS----
--SSKPNVH-------	-----SKIDKFF-----	------TKQEAQM----
--SSKPNVF-------	-----SKIATSL-----	------TKQEAQF----
--SSKPNTF-------	-----SKIANAVQVQKT	------TKQEAPI----
--SNKPNTT-------	-----SKIAESI-----	------TKQEANW----
---SKPNVY-W-----	-----SKIAEAY-----	------TKQEAGR----
---SKPNQY-S-----	-----SKIADSL-----	---THLTKQE-------
---SKPNQSY------	SGVFHSKLANAY-----	-----STKQETYTDKHY
---SKPNQSS------	---NTNKISNWFNIH--	-----STKQESL-----
---SKPNQSQ------	-NENAGKIATYLV----	----SQTKQELSANFL-
---SKPNQSN------	---NEGKISGYFWSI--	SPTYVLTKQERV-----
---SKPNQGY------	LLANTGKIQKYL-----	-SPDHYTKQERNR----
---SKPNQGM------	-GYFSGKVAQAQR----	--SNNLTKMEWLET---
---SKPNQF-H-----	GTYPVSKVASAF-----	---SNKTKQE-------
---SKPNMY-S-----	-----GKYQQYLQNYRA	---SNGTKQE-------
---SKPNMY-N-----	-----GKVTQALRGNWG	----SLTKQQHLDTEP-
---SKPNMY-A-----	-----GKVTERMTQALK	--SHPFSKQELREN---
---SKPNMF-M-----	-----GKVSNAY-----	---SHGTKQE-------
--SHKPNVF-------	-----GKVATAL-----	-SGIGHTKMELHR----
--SGKPNVW-------	-----GKVADYY-----	---QWQTKQE-------
--SGKPNLY-------	-----GKVADSFELPPG	---QNGTKQE-------
--SGKPNKW-------	-----GKLTEYLSRDAS	----QLTKQER------
--QGKPNQWANYFL--	-----GKLTDKL-----	---PNLTKQE-------
-PHKKPNQ--------	-----GKLSTYY-----	---PLKTKQE-------
----NVGQWPR-----	-----GKLSTFF-----	---PGQTKQE-------
---NKPNVF-G-----	-----GKLSSVMDGSKH	----NWTKQERWAVSA-
---NKPNQY-G-----	-----GKLSSFL-----	NVSFL-TKQELSR----
---NKPNQY-F-----	-----GKLSNLFDFYSK	-----NTKQETYYVLGS
---NKPNQF-Y-----	-----GKLSMAYATAIE	-----NTKQEIA-----
---NKPNMY-A-----	-----GKLSIYY-----	---NMLTKQE-------

---NKPNMSS------	-----GKLSESFFEKLT	----NLSKQERLQLSI-
---NKPNKF-M-----	-----GKLSEKL-----	---MKGTKQE-------
--MSKPNQW-------	-----GKLSDSINRSKM	----MGTKQEHLGPIR-
---MHPNTYP------	-----GKLSDMFDDPSR	---MDPTKQEKRSQI--
--MDKPNRW-------	-----GKLSDLLSPEGQ	-----LTRQESHDLTNG
--MCKPNMACADLT--	-----GKLSAAYESKVP	-----LTKQEYI-----
---LKPNMF-A-----	-----GKLDAYYSRNRP	-----LTKQETS-----
--LDKPNRY-------	-----GKLATAYSSQIS	-----LTKQETMGRVAG
----KSNQYPQ-----	-----GKLASAM-----	-----LTKQETG-----
----KSNQYPM-----	-----GKLAQAYQANLS	-----LTKQESR-----
----KSNQWPV-----	-----GKLADYL-----	-----LTKQERM-----
----KSNMYPS-----	-----GKIYNAI-----	-----LTKQENV-----
----KSNMYPQ-----	-----GKIVNYIEDAHN	-----LTKQENT-----
----KQNQYPA-----	-----GKIVNSL-----	-----LTKQENS-----
----KPNVY-PY----	-----GKIVDSLGQSSP	-----LTKQEMIVDSRF
----KPNVY-PS----	-----GKIVDSHVDMFR	-----LTKQEKAQTVPW
----KPNVY-PL----	-----GKITSTL-----	----L-TKQEIT-----
----KPNVWPT-----	-----GKITSQL-----	-----LTKQEGM-----
----KPNVWPR-----	-----GKITSMY-----	-LSNQYTRQEMLD----
----KPNVGPT-----	-----GKITQSL-----	----LQTKQEDWTPRS-
----KPNVF-PS----	-----GKITQAMNVSQR	----LPTKQEHYTFHQ-
----KPNTWPT-----	-----GKITNVF-----	--LPAHTKQEMRYL---
----KPNTWPQ-----	-----GKITNSL-----	-LNRAVTKQEMWP----
----KPNTTRI-----	-----GKITNAY-----	---LGKTKQE-------
----KPNTSKL-----	-----GKITNAL-----	KPADTYTKQQKF-----
----KPNTSAG-----	-----GKITKAL-----	---KAMTKQE-------
----KPNTNRY-----	-----GKITDSNTVMIT	----I-TKQELR-----
----KPNTNRV-----	-----GKITDSM-----	----I-TKQELA-----
----KPNTNRL-----	-----GKITDRH-----	----I-TKQEAN-----
----KPNTNRI-----	-----GKITDIMTLKRD	-----ITKQEALDTQIR
----KPNTNLS-----	-----GKITAAY-----	-ISIHATKQENFQ----
----KPNTNKT-----	-----GKITAAM-----	---IKATKQEIALLE--
----KPNTNKI-----	-----GKISYSM-----	---IGITKQE-------
----KPNTNAL-----	-----GKISVYNQIVHQ	HTYFAVTKQERL-----
----KPNSWASYPYLL	-----GKISTFL-----	----H-TKTELLDALVS
----KPNSFGS-----	-----GKISTFK-----	-----HTKQEMS-----
----KPNRV-PM----	-----GKISTAY-----	-----HTKQELR-----
----KPNRDAY-----	-----GKISSVMAHGDW	----H-TKQELL-----
----KPNQY-PT----	-----GKISSVF-----	HSPSLGTKMERA-----
----KPNQY-PL----	-----GKISSSLASTAN	----H-SKMELFESMSS
----KPNQY-PI----	-----GKISSMLLSDPQ	-HSDMVTKQEKWD----
----KPNQTRQ-----	-----GKISSIL-----	---HMETKQEKQIIW--
----KPNQTLS-----	-----GKISSAL-----	HLNLLSTKQEMH-----
----KPNQTLL-----	-----GKISNVL-----	---GYFTKQE-------
----KPNQTLK-----	-----GKISNVF-----	---GWLTKQEVLTSV--
----KPNQTKY-----	-----GKISNTF-----	GVSHSYTKQELV-----
----KPNQSNY-----	-----GKISNSYTTHTM	---GVLTKQE-------
----KPNQSNG-----	-----GKISNLL-----	GTPDARTKQETW-----
----KPNQM-PS----	-----GKISHKL-----	----G-TKQELVMLRTV
----KPNQL-PW----	-----GKISHFM-----	----G-TKQELLEHGGR
----KPNQGPT-----	-----GKISHAY-----	---GSFTKQE-------

----KPNQGPG-----	-----GKISEVL-----	----GMSHQERYELQW-
----KPNQF-PR----	-----GKISEAS-----	----GFTKQEKWSAIH-
----KPNQEGT-----	-----GKISEAK-----	GDTGRFTKQQMT-----
----KPNMY-PL----	-----GKISDRIKFDDG	-----FTKQERF-----
----KPNMW-PG----	-----GKISASY-----	-----FTKMERA-----
----KPNMR-PS----	-----GKINQSL-----	-----ETKQERI-----
----KPNMF-PR----	-----GKINNWY-----	---ESRTKQE-------
----KPNMF-PG----	-----GKINNAL-----	ESQSHMTKQEVH-----
----KPNLY-PY----	-----GKINNAF-----	---ERGTKQE-------
----KPNLY-PL----	-----GKINDFM-----	---EGLTKQE-------
----KPNLY-PI----	-----GKILTHL-----	--EGFLTKQERSSY---
----KPNLY-PA----	-----GKILDAM-----	----DWTKQEMLMGHP-
----KPNLWPQ-----	-----GKIHNAM-----	--DRLSTKQEMWRP---
----KPNLL-PL----	-----GKIHNAL-----	--DGKYTKQEHYTV---
----KPNLF-PR----	-----GKIHDVFSHDTI	----AWTKQEL------
----KPNLF-PQ----	-----GKIHDSL-----	-----ATKQEVY-----
----KPNLF-PL----	-----GKIGSFLGGGHI	-----ATKQEIL-----
----KPNKWPD-----	-----GKIGQYFSEYAT	-ASESPTKQQFVD----
----KPNKF-PA----	-----GKIFNTL-----	---APLTKQE-------
----KPNFL-PK----	-----GKIEWTM-----	---ALDTKQQTRDLV--
----KPNCPSC-----	-----GKIESHF-----	---AEMTKQESILQR--
----KPNAY-PA----	-----GKIEAVYTNPYK	
----KMNQWPP-----	-----GKIDYFI-----	
---KKPNMY-Y-----	-----GKIDYAL-----	
---KKPNMY-A-----	-----GKIDTFL-----	
---KKPNIY-N-----	-----GKIDSYF-----	
----KENRWPM-----	-----GKIDSFIRVEHG	
----KENQWPS-----	-----GKIDNVLLKGLR	
----KANVYPM-----	-----GKIDALL-----	
----KANVYPG-----	-----GKIATSL-----	
----KANVWPL-----	-----GKIATRL-----	
----KANVFPT-----	-----GKIAQQL-----	
----KANSYPQ-----	-----GKIAQEL-----	
----KANSFGS-----	-----GKIAHYY-----	
----KANRDPN-----	-----GKIAHAYRLGDA	
----KANQSKI-----	-----GKIAEKM-----	
----KANQGPL-----	-----GKIADLG-----	
----KANQF-PW----	-----GKIADKH-----	
----KANQERT-----	-----GKIADAI-----	
----KANMYPR-----	-----GKIAAQY-----	
----KANMYPF-----	--GFAGKIASTFVD---	
----KANMWPS-----	----DSKIADFYFTKM-	
----KANMWPN-----	-----DKISHAMMTHTR	
----KANMWPA-----	-AYPSSKIFSVLE----	
----KANMGPR-----	-----AKISNAL-----	
----KANMGPF-----	-----AKISDVL-----	
----KANKYPS-----	-----AKIANVL-----	
---IKPNQF-R-----		
-IAHKPNQGWWIH---		
--HTKSNQWYPFQM--		
--HSKSNQF-------		

--HSKMNQY-------
--HLKPNQV-------
---HKPNMR-H-----
---HKPNMH-G-----
---HKANQFL------
--HIKPNMWNPRPN--
--HHKANLY-------
--GSKANLF-------
--GPKANQW-------
---GKPNTY-Y-----
--GKKPNTY-------
--GFKPNMF-YYPEL-
--GAKPNQW-------
--GAKANQFYLTYA--
--GAKANLFWSSPT--
---FKPNVY-T-----
---FKPNQY-L-----
---FHPNTYN------
---FHPNTWN------
--DSKPNRF-------
--DPKPNSSDYWYF--
-ASCKPNTSWCRN---
---AKPNVY-W-----
---AKPNVY-DDVVIA
---AKPNQWP------
---AKPNMY-R-----

Binding of IgG antibodies to epitope-like peptides

Suppl. Table 3: Demographic and serologic features of individuals included in the study. Six individual sera were combined into one sample, resulting in the end in six pooled sera.

Patient nr.	Sex	Age	sIgE wasp	sIgE Ves v 5	Label
1	F	32	1.06	3.6	#1
2	F	35	6.13	20.3	
3	M	52	9.82	56	
4	M	25	21.1	43.5	
5	M	39	4.53	12.5	
6	F	58	9.54	47.6	
7	F	30	27.2	67	#2
8	M	66	11	0.971	
9	M	45	8.21	36.3	
10	F	43	9.87	12.9	
11	M	36	7.63	33.2	
12	M	32	1.65	19.9	
13	F	23	18.7	65.7	

14	M	49	6.58	4.92	#3
15	F	59	6.78	12.2	
16	F	56	5	24.7	
17	M	42	2.79	15	
18	F	33	5.35	17.6	
19	M	26	26.6	57.7	#4
20	M	48	0.84	0.656	
21	F	54	67.8	6.15	
22	F	35	2.07	0.9	
23	M	19	11.5	5.24	
24	M	21	2.9	1.54	
25	M	62	4.43	33.5	#5
26	F	60	87.4	0.48	
27	F	37	8	6.6	
28	F	24	2.24	0.5	
29	F	39	1.26	4.51	
30	M	66	22.3	1.84	
31	F	49	19.8	15.7	#6
32	F	19	3.11	0.53	
33	F	34	4.34	8.86	
34	F	39	4.11	0.76	
35	M	32	5.24	3.68	
36	F	25	1.12	2.51	

Suppl. Table 4: Sequences of linear peptides subjected to microarray analysis and the respective immunofluorescence intensities from six groups of individuals. Negative control peptides are designated in bold.

Sequence	#1	#2	#3	#4	#5	#6	MEDIAN
HTKQELL	50864.33	24233.33	41363.67	62352.67	44506.67	40949.33	42935.17
YSSLKPNKYAVW	15893.33	33830.00	19442.00	36808.67	31678.33	41532.33	32754.17
GFKPNMFYYPEL	19835.33	20461.00	21825.67	43457.67	31935.67	43782.67	26880.67
KANQFPW	12957.67	29566.67	15048.00	27505.00	24024.33	28984.67	25764.67
GKIGSFLGGGHI	14442.33	17750.33	20801.00	29874.00	27154.67	32336.33	23977.83
DPKPNSSDYWYF	12557.00	14137.00	16060.33	32262.67	30039.00	36881.67	23049.67
GKIDSYF	12697.67	14531.33	15807.33	32325.33	27926.33	32145.67	21866.83
IAHKPNQGWWIH	11793.33	60922.33	14252.67	23686.33	19107.33	22857.33	20982.33
GFAGKIASTFVD	9222.33	20327.33	12665.33	21946.33	19958.67	44890.67	20143.00
GKIDYFI	11930.33	12806.00	14226.67	27226.33	25027.00	27157.00	19626.83
QGKPNQWANYFL	11452.00	11568.33	15335.67	22906.33	20535.33	25952.33	17935.50
HMETKQEKQIIW	9398.67	15792.00	11499.00	22332.33	18189.67	22506.33	16990.83
GKIDSFIRVEHG	9480.67	10240.00	12590.33	30291.33	20583.67	26616.00	16587.00
GKIGQYFSEYAT	7821.67	11660.33	9955.00	23605.33	21245.33	22521.67	16452.83
GKISSVMAHGDW	9220.00	14373.33	15209.67	15568.33	15278.33	18010.33	15244.00
STKQETYTDKHY	10782.67	9859.00	12438.67	18233.33	17596.00	18598.33	15017.33
LLANTGKIQKYL	8964.33	10023.67	11715.33	21481.33	17429.67	23338.00	14572.50
TKQELPY	8870.00	9407.00	10504.67	20470.33	16003.67	20255.67	13254.17
GKITNAY	8690.33	8751.33	10240.33	34280.33	15723.33	19068.00	12981.83
NKPNQYF	9383.00	8121.67	10404.33	16407.00	14739.00	22454.33	12571.67
ATKQEVY	7378.00	7184.33	9099.33	16262.33	12392.67	16219.00	10746.00
LDKPNRY	8207.33	6957.33	8587.67	14372.67	12801.33	17421.00	10694.50
GKIFNTL	7059.00	7945.00	8433.67	13486.00	12239.67	14953.33	10336.67
SHKPNVF	8310.67	6121.00	9111.00	12728.33	11525.33	12021.00	10318.17
KPNVYPL	7797.00	7104.67	8929.67	15093.67	11119.00	11305.67	10024.33
AKPNVYDDVVIA	6681.67	5528.67	7947.33	11029.67	11802.33	12952.00	9488.50

TKQEANW	5694.33	8613.67	7399.67	14955.67	10178.67	12699.00	9396.17
SGKPNVW	6769.33	13646.33	7107.00	9262.33	8757.33	8907.33	8832.33
GKISDRIKFDDG	5969.67	6664.00	6777.00	37386.67	10554.33	13763.33	8665.67
TNVYTKQESMIM	4017.00	6947.00	5394.67	11490.00	9124.00	11673.67	8035.50
GKIVDSHVDMFR	6426.67	5372.67	6721.33	11688.67	9311.33	13218.33	8016.33
GKITSMY	6966.33	5959.67	7051.33	8656.33	9590.67	8544.33	7797.83
GKITDIMTLKRD	5075.67	16964.00	5727.33	8243.00	7099.33	9680.67	7671.17
KPNQYPI	5665.00	5974.00	6570.33	9347.67	7780.33	9155.00	7175.33
GKINDFM	6002.00	4528.33	6702.67	9183.00	7255.00	8149.00	6978.83
ITKQELR	5578.33	5141.00	5440.33	11328.00	8181.00	12101.33	6879.67
LNRAVTKQEMWP	4538.00	4550.67	5726.67	7672.00	10114.00	10221.67	6699.33
**QQLNIPP**	**4708.67**	**4575.33**	**6046.67**	**9009.00**	**7150.33**	**8789.33**	**6598.50**
VKPNLYPSWNMS	4794.67	4511.33	7407.00	6536.67	5926.00	6510.67	6218.33
VKPNLYPSNDPI	5416.67	3510.33	8521.67	6965.33	5449.67	7018.00	6207.50
LPAHTKQEMRYL	6051.33	3306.00	4620.33	7957.33	6207.00	11504.67	6129.17
NWTKQERWAVSA	4982.67	4206.67	4978.67	8548.33	7004.33	9068.67	5993.50
ESQSHMTKQEVH	3930.67	13084.33	3943.33	6632.67	5040.33	6721.00	5836.50
ITKQEALDTQIR	3671.67	4948.33	4335.67	8069.33	6537.33	8465.33	5742.83
HIKPNMWNPRPN	4107.67	63709.67	3577.67	5861.00	6647.67	5515.00	5688.00
VTKQEYM	4498.33	3714.00	4835.67	8054.67	6456.67	8527.00	5646.17
GKISSAL	3788.67	3483.00	5181.67	8113.00	5228.33	7410.33	5205.00
KPNQSNY	4196.67	4128.00	4363.00	7250.67	5838.67	7692.00	5100.83
EGLTKQE	3950.00	3641.67	4675.67	6898.00	5434.67	6154.00	5055.17
GKIADLG	4069.67	4407.67	4462.33	6599.67	5614.33	6894.33	5038.33
GKIAQEL	4415.33	3899.33	4587.67	20145.67	5814.33	5445.00	5016.33
TTKQERL	4366.67	4240.33	3943.00	50581.33	5665.33	5585.33	4976.00
ITKQELA	3444.00	3606.00	4113.33	7407.00	5752.67	7991.00	4933.00
KPNMYPL	4355.67	3428.67	4611.00	7649.33	4924.33	5879.33	4767.67
WGMTKQE	3747.67	4230.33	4124.67	8746.00	5188.00	7559.33	4709.17
MGTKQEHLGPIR	5220.33	3405.00	3274.00	5458.00	4196.33	6418.33	4708.33

KANQERT	3844.67	3296.33	4141.00	6837.33	5256.67	6672.00	4698.83
LTKQESR	3659.00	3045.00	3378.00	6155.00	4900.33	7515.33	4279.67
TKQEMRL	4116.33	2401.00	4113.00	8252.00	4406.67	7836.00	4261.50
ERGTKQE	2589.00	2614.33	4500.00	5111.00	3982.67	5783.67	4241.33
GKITNAL	3274.67	2989.67	3805.67	6903.33	4576.67	6123.33	4191.17
GKITQAMNVSQR	3494.33	2764.00	4344.00	8113.67	3961.67	5483.67	4152.83
KANMWPS	4060.67	3403.00	3801.00	5167.00	4114.67	5392.33	4087.67
KANKYPS	3822.33	3283.33	3318.00	4653.33	4196.00	4766.33	4009.17
NMLTKQE	2148.33	2755.67	2379.67	6102.33	5017.67	5954.00	3886.67
AEMTKQESILQR	3877.00	3443.00	3434.33	5500.00	3599.33	6981.33	3738.17
TKQEGRT	3274.67	2513.00	3524.00	5568.67	3916.33	4318.67	3720.17
**AHRVQTA**	**3337.33**	**2839.67**	**2986.33**	**5260.33**	**4102.67**	**5303.33**	**3720.00**
TKQEAPI	2621.00	2692.00	3365.67	5266.00	3544.33	5422.33	3455.00
PNLTKQE	2389.17	2714.83	2590.50	5403.83	4120.17	5443.50	3417.50
**NLLMSHA**	**3125.67**	**2462.67**	**3309.67**	**4847.33**	**3314.00**	**4793.67**	**3311.83**
GKIATSL	2911.00	2731.00	3102.33	5714.00	3428.67	3865.67	3265.50
ITKQEAN	1907.67	17160.33	2014.33	3587.67	2858.67	4258.67	3223.17
WKPNQAPFPAPM	2402.33	2434.00	2778.00	5224.67	3519.00	4762.67	3148.50
LTKQETS	2896.67	2248.67	2509.67	4220.33	3280.33	5371.67	3088.50
LTKQEGM	2325.67	2291.33	2389.67	4186.67	3498.00	5281.67	2943.83
GKITNSL	2486.00	2135.33	2701.33	5482.00	2783.33	4399.33	2742.33
SNGTKQE	2015.33	2232.00	2036.67	3944.67	3110.67	4073.67	2671.33
VPKPNVGMWGPV	2742.00	2140.33	2466.33	4536.67	2349.33	3443.67	2604.17
LTKQERM	2731.67	1961.33	1946.67	3235.67	2437.33	3840.33	2584.50
GKISNSYTTHTM	2327.33	1741.33	2041.00	3984.33	2648.00	4211.33	2487.67
GKIVDSLGQSSP	1991.67	1727.33	1630.67	2828.00	2362.00	4758.33	2176.83
VKAHVSPTKQEM	1760.67	1562.67	1605.00	2052.00	1801.67	2523.00	1781.17

Suppl. Table 5: Sequences of cyclic peptides subjected to microarray analysis and the respective immunofluorescence intensities from six groups of individuals. Negative control peptides are designated in bold.

Sequence	#1	#2	#3	#4	#5	#6	MEDIAN
CGFKPNMFYYPELC	23809.33	25518.00	31407.00	55694.00	48075.33	56814.00	39741.17
CYSSLKPNKYAVWC	19036.00	23484.67	23492.33	45662.00	42490.67	53047.67	32991.50
CDPKPNSSDYWYFC	15191.00	17652.33	19554.00	39612.33	44876.00	42230.00	29583.17
CKANQFPWC	18027.50	20680.50	21665.83	42247.17	33746.50	45315.83	27706.17
CGKIDSYFC	12864.00	17037.33	17787.33	40936.67	34880.33	38725.33	26333.83
CVKPNLYPSNDPIC	24709.00	12940.67	24388.00	25614.00	21818.00	24639.33	24513.67
CGKIDSFIRVEHGC	13109.67	14898.67	16240.33	41205.67	30876.00	37868.00	23558.17
CNKPNQYFC	13308.83	15986.83	16892.17	37767.83	28445.17	39057.50	22668.67
CITKQELRC	12912.00	16251.00	15836.67	29175.33	28793.33	31192.67	22522.17
CGKIDYFIC	11840.67	16604.33	15350.33	34107.00	27763.00	31194.33	22183.67
CQGKPNQWANYFLC	13234.33	16274.33	16147.00	32148.33	27909.67	33087.00	22092.00
CGKIFNTLC	14278.67	14749.67	15036.00	28523.33	26337.67	31639.67	20686.83
CGKITNAYC	12795.33	14760.33	16577.67	42467.00	24613.33	33368.33	20595.50
CKPNVYPLC	15012.00	17073.67	16029.00	34217.00	23719.33	32478.00	20396.50
CSTKQETYTDKHYC	13593.67	13867.67	15466.33	25523.67	23338.33	27379.33	19402.33
CTKQELPYC	11666.67	13388.00	14509.00	32360.33	22800.67	29631.00	18654.83
CAKPNVYDDVVIAC	10651.00	11312.33	13409.00	23291.33	22877.33	25964.33	18143.17
CHMETKQEKQIIWC	11429.00	12541.67	14500.33	31837.33	21016.33	31333.67	17758.33
CGKIGSFLGGGHIC	12813.00	14147.00	16898.67	20978.00	17407.67	21757.00	17153.17
CKPNQYPIC	13001.00	12660.00	13367.67	22773.00	19474.00	29588.00	16420.83
CGKIGQYFSEYATC	8311.00	11827.33	11641.00	24939.33	20885.67	24301.33	16356.50
CSGKPNVWC	13084.67	12191.33	13221.33	21903.67	19287.33	29545.67	16254.33
CIAHKPNQGWWIHC	12765.67	15068.67	12969.33	20904.33	17176.00	30667.33	16122.33
CITKQELAC	9057.00	11169.33	11482.67	23597.00	20357.33	22214.33	15920.00
CGKISSVMAHGDWC	9801.00	12618.33	15523.00	16136.00	16715.67	18131.00	15829.50

CHTKQELLC	10408.67	11443.67	12196.33	22735.67	19211.67	22129.00	15704.00
CTKQEANWC	10809.00	12504.33	12399.00	26462.33	18486.33	23898.33	15495.33
CGKIAQELC	10818.00	9768.67	12320.00	18450.33	20428.33	20625.33	15385.17
CGKINDFMC	10212.00	15381.33	10756.67	16221.00	15271.67	24701.33	15326.50
CLDKPNRYC	11708.67	10910.33	11943.67	18332.33	18175.67	31521.00	15059.67
CGFAGKIASTFVDC	8724.33	11170.67	10855.67	21678.33	17986.67	22607.67	14578.67
CATKQEVYC	7524.67	10133.33	9639.33	23730.33	18896.00	19409.67	14514.67
CKPNQSNYC	8603.33	8981.33	11262.67	14877.00	15579.33	22123.00	13069.83
CLLANTGKIQKYLC	9188.00	11335.67	9160.67	16737.67	13711.33	19309.67	12523.50
CVKPNLYPSWNMSC	12755.67	6277.33	14113.67	12218.33	10107.33	17261.00	12487.00
CGKIADLGC	8668.67	11256.33	9638.33	14269.67	13185.67	16966.00	12221.00
CSHKPNVFC	11004.33	9739.00	9599.33	17200.00	13385.33	18392.33	12194.83
CVTKQEYMC	7473.67	9775.00	8949.00	20472.33	14047.00	16258.33	11911.00
CGKISDRIKFDDGC	8343.67	8428.67	10073.00	24346.67	13442.00	18172.67	11757.50
**CQQLNIPPC**	**8041.33**	**8028.67**	**9297.33**	**18847.00**	**13467.00**	**15584.67**	**11382.17**
CTKQEAPIC	10552.17	7393.83	20247.83	13522.50	9185.83	12014.83	11283.50
CKPNMYPLC	9389.33	8886.00	8440.67	12129.67	10559.00	17505.00	9974.17
CWGMTKQEC	5525.67	6472.00	6486.67	13734.00	24735.33	12260.67	9373.67
CEGLTKQEC	6829.00	6207.00	7580.67	10677.00	15031.67	17224.33	9128.83
CNWTKQERWAVSAC	4945.00	6033.33	6749.00	12674.67	10281.33	14214.00	8515.17
CLTKQETSC	5967.67	6571.00	6481.33	12539.33	9755.00	11363.33	8163.00
CGKITSMYC	6377.00	7520.67	6942.67	8105.00	7955.67	10487.33	7738.17
CLTKQESRC	6886.33	5901.67	6170.00	9863.33	8331.67	9593.00	7609.00
CPNLTKQEC	5042.33	4864.00	5296.00	8952.00	13939.00	10858.33	7124.00
CTNVYTKQESMIMC	4387.33	5439.00	5896.00	11507.33	8230.67	14572.33	7063.33
CGKITDIMTLKRDC	6487.33	5727.00	6932.67	8122.00	7049.00	9983.33	6990.83
CGKIVDSHVDMFRC	4040.00	5361.00	5315.33	10774.00	8535.00	14103.00	6948.00
CERGTKQEC	4592.00	3396.00	3435.50	27715.00	9588.33	9123.33	6857.67
CTTKQERLC	6091.00	4635.00	4935.00	6593.00	6523.67	7091.67	6307.33
CNMLTKQEC	4539.00	4294.67	4639.67	7881.00	18377.67	8943.33	6260.33

CITKQEALDTQIRC	6026.00	5536.33	4856.00	6649.67	6437.00	7435.67	6231.50
CLNRAVTKQEMWPC	6199.00	5241.67	5292.00	6185.33	6303.33	10325.00	6192.17
CGKITQAMNVSQRC	5259.00	4592.67	5723.67	8868.33	6496.00	8965.67	6109.83
CVPKPNVGMWGPVC	6965.00	3909.00	5228.00	7799.33	4435.00	6925.00	6076.50
CKANMWPSC	6492.83	4725.50	4659.83	6067.50	5899.17	16057.50	5983.33
CGKISSALC	4951.00	4654.00	5856.33	6560.67	5964.67	6971.67	5910.50
CGKITNALC	5091.00	3812.00	6292.00	5494.33	5991.67	9193.67	5743.00
CITKQEANC	4547.00	5001.67	4193.00	7481.00	6390.00	10100.33	5695.83
CKANKYPSC	4872.00	5988.33	4353.33	5824.00	5236.67	14626.00	5530.33
CESQSHMTKQEVHC	5603.67	3639.00	3791.33	6346.67	5052.33	7702.00	5328.00
CGKITNSLC	4945.67	3645.67	5514.33	5011.67	6624.33	10958.67	5263.00
CLPAHTKQEMRYLC	4987.33	4667.67	4128.00	5804.00	5371.33	5527.00	5179.33
CSNGTKQEC	2783.33	2728.33	2152.00	6101.67	8836.00	5959.00	4371.17
CAEMTKQESILQRC	5236.67	2303.67	3450.67	5826.67	2999.67	5528.67	4343.67
CKANQERTC	4009.67	3579.33	4087.33	6452.00	4470.00	6838.67	4278.67
**CAHRVQTAC**	**4421.00**	**2351.00**	**2831.67**	**5411.67**	**3631.33**	**4647.00**	**4026.17**
CGKIVDSLGQSSPC	5009.00	3596.67	4056.00	3959.67	3706.33	11784.00	4007.83
CTKQEGRTC	3545.67	2842.67	3234.67	5282.67	2991.33	5447.00	3390.17
**CNLLMSHAC**	**3973.33**	**2137.33**	**3121.33**	**3607.67**	**2239.67**	**5498.33**	**3364.50**
CHIKPNMWNPRPNC	5293.33	2280.00	1956.00	4493.67	2791.00	3888.67	3339.83
CLTKQEGMC	3494.00	2838.00	2765.00	4226.33	3140.67	5473.00	3317.33
CWKPNQAPFPAPMC	2638.67	5270.00	2252.67	3905.33	2396.67	4794.33	3272.00
CTKQEMRLC	4715.83	1927.50	2134.17	3258.83	2622.17	4056.17	2940.50
CMGTKQEHLGPIRC	5767.33	1772.67	1799.00	3661.67	2371.00	3401.00	2886.00
CLTKQERMC	4505.50	1887.83	1628.17	3404.17	2034.83	3867.50	2719.50
CGKIATSLC	1890.33	2422.00	2620.33	2463.00	2350.33	3542.33	2442.50
CGKISNSYTTHTMC	2581.67	1960.67	1478.67	16783.00	2404.67	2452.67	2428.67
CVKAHVSPTKQEMC	2677.33	1705.67	1379.67	2248.67	1874.00	1939.00	1906.50
